# Transcriptome Profiling and Cytological Assessments for Identifying Regulatory Pathways Associated With Diorcinol N-Induced Autophagy in A3 Cells

**DOI:** 10.3389/fphar.2020.570450

**Published:** 2020-10-15

**Authors:** Xiao-Long Yuan, Xiu-Qi Li, Kuo Xu, Xiao-Dong Hou, Zhong-Feng Zhang, Lin Xue, Xin-Min Liu, Peng Zhang

**Affiliations:** ^1^ Tobacco Research Institute, Chinese Academy of Agricultural Sciences, Qingdao, China; ^2^ Graduate School of Chinese Academy of Agricultural Sciences, Beijing, China; ^3^ Wannan Tobacco Group Company Limited, Xuancheng, China

**Keywords:** acute lymphocytic leukemia, autophagy, diorcinol N, G1/S phase arrest, transcriptome profiling

## Abstract

Fungal secondary metabolites serve as a rich resource for exploring lead compounds with medicinal importance. Diorcinol N (DN), a fungal secondary metabolite isolated from an endophytic fungus, *Arthrinium arundinis*, exhibits robust anticancer activity. However, the anticancer mechanism of DN remains unclear. In this study, we examined the growth-inhibitory effect of DN on different human cancer cell lines. We found that DN decreased the viability of A3 T-cell leukemia cells in a time- and concentration-dependent manner. Transcriptome analysis indicated that DN modulated the transcriptome of A3 cells. In total, 9,340 differentially expressed genes were found, among which 4,378 downregulated genes and 4,962 upregulated genes were mainly involved in autophagy, cell cycle, and DNA replication. Furthermore, we demonstrated that DN induced autophagy, cell cycle arrest in the G1/S phase, and downregulated the expression of autophagy- and cell cycle-related genes in A3 cells. By labeling A3 cells with acridine orange/ethidium bromide, Hoechst 33,258, and monodansylcadaverine and *via* transmission electron microscopy, we found that DN increased plasma membrane permeability, structural disorganization, vacuolation, and autophagosome formation. Our study provides evidence for the mechanism of anticancer activity of DN in T-cell leukemia (A3) cells and demonstrates the promise of DN as a lead or even candidate molecule for the treatment of acute lymphoblastic leukemia.

## Introduction

Acute lymphoblastic leukemia (ALL) is a hematological malignancy associated with uncontrolled proliferation and transformation of lymphoid progenitor cells within the bone marrow (Soulier and Cortes, 2015). Over the past few decades, the revolution in tumor cell biology and chemotherapeutic strategies coupled with high-throughput sequencing has led to significant improvement in outcomes for pediatric patients (Hunger and Mullighan, 2015). The survival rate of leukemia patients can reach 90% in children under 14 years of age, while it can decrease to 40% in adults between 25 and 64 years of age and to almost 15% in adults aged over 65 (Curran and Stock, 2015; Hunger and Mullighan, 2015; Kansagra et al., 2018). The poor prognosis for the elderly is because of the ease of metastasis to different organs and frequent relapse (Aldoss and Stein, 2018). The most difficult problem is in the treatment of hematologic malignancies is that few treatments show the desired therapeutic efficacy or achieve complete responses (Phelan and Advani, 2018). Further, the use of localized surgery and radiation approaches may not always be possible to prevent the dissemination of tumor cells (Malard and Mohty, 2020). To date, chemotherapy is the most preferred treatment of choice for these patients (Paul et al., 2017). Therefore, there is an urgent need to develop novel therapies that overcome resistance to the currently administered anticancer drugs for ALL cells.

Secondary metabolites, especially those produced by endophytic fungi, have been demonstrated to be rich sources of not only anticancer lead compounds with high potential against ALL cells, but have also contributed significantly to the discovery of novel drugs (Deshmukh et al., 2019). For example, Ophiobolins A, B, C, and K, obtained from three fungal strains in the Aspergillus section Usti, could reduce leukemia cell viability and induce cell apoptosis at nanomolar concentrations. Secalonic acid D, isolated as a secondary metabolite of the mangrove endophytic fungus No ZSU44, exhibited potent cytotoxicity to ALL cells. Further studies have indicated that secalonic acid D led to cell cycle arrest of G1 phase related to the downregulation of c-Myc *via* activation of GSK-3β, followed by degradation of β-catenin. Consequently, it is necessary to explore more secondary metabolites in endophytic fungi and to investigate their potential anticancer activity.

Diorcinols are prenylated diphenyl ether derivatives that are isolated from numerous endophytic fungi and possess various biological activities. For example, diorcinol D, which was isolated from an endolichenic fungus, showed fungicidal activity against *Candida albicans* by destroying the cytoplasmic membrane and generating reactive oxygen species (ROS) (Li et al., 2015). Diorcinol J, which was produced by co-cultivation of marine fungi, *Aspergillus sulphureus* and *Isaria feline*, can induce the expression of heat shock protein (Hsp70) in Ehrlich ascites carcinoma cells (Zhuravleva et al., 2016). Recently, in our ongoing search for structurally diverse metabolites with novel cytotoxic mechanisms, a new prenylated diphenyl ether, diorcinol N (DN), was isolated and identified from an endophytic fungus *Arthrinium arundini*, which was collected from fresh leaves of *Nicotiana tabacum* L. (Zhang et al., 2018). DN displayed promising cytotoxicity against the human THP-1 monocytic cell line in a cytotoxic assay (Li et al., 2018).

Thus, DN appears to be a potential candidate for blood cancer treatment and can be used as a lead for the development of novel, targeted anti-leukemia drugs. In this study, we performed cell-based assays and transcriptome profiling to investigate the anticancer mechanism of DN. First, we studied the effects of DN on the viability of selected human cancer cell lines. Transcriptome analysis was used to analyze DN-regulated genes and related signaling pathways that are responsible for growth and autophagy in A3 cells. In addition, the molecular mechanism of growth inhibition and autophagy induction by DN in this cell line was investigated *via* ultrastructural observation, flow cytometry, and quantitative reverse-transcription polymerase chain reaction (qRT-PCR).

## Materials and Methods

### Chemicals and Fungal Material

High-performance liquid chromatography (HPLC) was performed using a Waters ultra-performance liquid chromatography-class system equipped with a C_18_ column (1.6 μm, 2.1 × 50 mm) and a photodiode array detector. The chromatographic conditions were as follows: mobile phase: 10% methanol (MeOH), 0–5 min; 10%–100% MeOH, 5–35 min; 100% MeOH, 35–45 min; 100%–10% MeOH, 45–50 min; 10% MeOH, 50–60 min; flow rate: 1 ml/min; ultraviolet detection: 235 nm. High-resolution electrospray ionization mass spectrometry (HRESIMS) data were obtained with a Thermo Scientific LTQ Orbitrap XL spectrometer (Thermo Scientific, Waltham, MA, USA). The ^1^H, ^13^C, and two-dimensional nuclear magnetic resonance (NMR) spectra were measured using an Agilent DD2 spectrometer (500 and 125 MHz, respectively) (Agilent, Santa Clara, CA, USA). Open column chromatography was performed using silica gel (200−300 mesh, Qingdao Haiyang Chemical Factory, Qingdao, China), Lobar LiChroprep RP-18 (Merck, Darmstadt, Germany), and Sephadex LH-20 (Merck). All solvents used for HPLC, HRESIMS, and NMR analyses were of analytical grade (purchased from Merck, Darmstadt, Germany). The fungal strain, *A. arundinis*, was previously isolated from fresh leaves of *Nicotiana tabacum* L., with the GenBank number MK182939 and CGMCC number 14792 (Zhang et al., 2018).

### Cell Cultures

All cell lines used in this study were purchased from the Chinese Academy of Sciences Committee on Type Culture Collection Cell Bank (Shanghai, China) and then conserved in the Tobacco Research Institute of Chinese Academy of Agricultural Sciences. The human lymphoblastic leukemia Jurkat and A3 cell lines and human lung cancer HCC827 cell lines were cultured using Roswell Park Memorial Institute (RPMI) 1640 medium (RPMI-1640; #A1049101, Invitrogen, Carlsbad, CA, USA) containing 10% fetal bovine serum (FBS; #16140071, Gibco, Carlsbad, CA, USA). The human breast cancer cell lines, MCF-7 and MDA-MB-231, human cervical cancer cell line, HeLa, and human prostate cancer cell lines DU-145 and PC-3, were cultured in Minimum Essential Medium (MEM; #10370021, Invitrogen, Carlsbad, CA, USA) supplemented with 10% FBS. The human lung cancer cell line, A549, was maintained in Ham’s F-12K (Kaighn’s) Medium (#21127022, Invitrogen, Carlsbad, CA, USA) supplemented with 10% FBS. We isolated peripheral blood mononuclear cells (PBMCs) *via* density-gradient centrifugation using a Lymphocyte Separation Solution (NakalaiTesque, Kyoto, Japan). Subsequently, we harvested the PBMCs by centrifugation at 1,500 rpm for 10 min at 22°C and then resuspended them in RPMI 1640 with 10% FBS (Gibco). All cells were cultured in a humidified atmosphere containing 5% CO_2_ at 37°C.

### Purification of DN From *A. arundinis*

**Figure 1:**
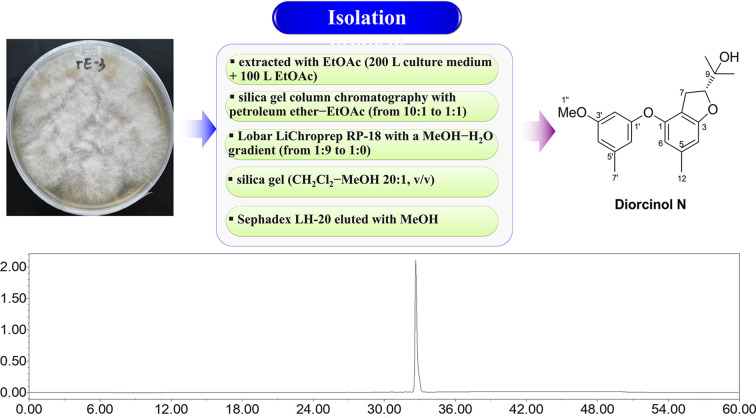
Isolation flowchart, chemical structure, and high-performance liquid chromatography (HPLC) analysis of Diorcinol N (DN).

The general isolation procedure for DN is shown in **Figure 1**. Details of fermentation, extraction, and isolation procedures have been reported previously (Zhang et al., 2018). Briefly, to obtain sufficient amounts of DN, large-scale fermentation was performed with the productive strain, *A. arundinis*, in liquid Potato Dextrose Broth medium. The broth (approximately 200 L) was extracted exhaustively with 100 L of ethyl acetate (EtOAc), which was evaporated to yield 86 g of residue. The residue was subjected to silica gel column chromatography with a mobile phase consisting of mixed petroleum ether (PE)-EtOAc (from 10:1 to 1:1, v/v) to yield four fractions (Frs. 1–4). Fr. 4 (8.0 g), eluting with PE-EtOAc 1:1, was applied to Lobar LiChroprep reverse phase (RP)-18 with a MeOH-H_2_O gradient (from 1:9 to 1:0) to give three subfractions (Frs. 4.1–4.3). Fr. 4.3 was separated using silica gel (dichloromethane-MeOH 20:1), followed by Sephadex LH-20 (MeOH) to yield DN (120 mg). Finally, DN with a purity of more than 98% was obtained and dissolved in dimethyl sulfoxide (DMSO) to prepare a 100-mM stock solution. Since a large number of experiments have shown that 0.1% DMSO is not cytotoxic to different tumor cells (Chou et al., 2018; Chiang et al., 2019; Song et al., 2020), DN was diluted further in cell culture media for use in the treatment of cells, so that the final concentration of DMSO in wells was 0.1% at the highest concentration of DN used in the study. Viability assays showed that 0.1% DMSO was non-toxic to cells (data not shown). All solvents (EtOAc, PE, dichloromethane, MeOH, and DMSO) used for column chromatography were of chemical grade (purchased from Sinopharm Chemical Reagent Co., Ltd, Shanghai, China).

### Cell Viability Analysis

The effects of DN on cell viability were investigated by using the Cell Counting Kit-8 (CCK-8) assay. Briefly, 3.0 × 10^4^ cells/well were seeded in 96-well plates for 24 h of incubation. Then, the cells were co-cultured for 24, 48, and 72 h with various concentrations of DN (0, 3.125, 6.25, 12.5, 25, 50, and 100 μM), after which cell viability was assessed using the CCK-8 Cell Proliferation and Cytotoxicity Assay Kit (#CA1210, Solarbio, Beijing, China). Absorbance was measured using a microplate spectrophotometer (Multiskan GO, Thermo Scientific, Waltham, MA, USA) at 490 nm.

### Cell Cycle Analysis

After treatment with 6.25, 12.5, and 25 μM DN for 48 h, 1 × 10^6^ A3 cells/ml were collected, washed three times with phosphate-buffered saline (PBS), and fixed overnight by incubation with 70% ethanol. The fixed cells were stained using a DNA Content Quantitation Assay Kit (#CA1510, Solarbio, Beijing, China) for cell cycle analysis. The test data were analyzed using Multicycle (Phoenix Flow Systems, San Diego, CA).

### Fluorescence Microscopy

The morphology of A3 cells was observed by staining with acridine orange/ethidium bromide (AO/EB; Sigma-Aldrich Corp., St. Louis, MO, USA) and monodansylcadaverine (MDC; ab139484; Abcam), as described previously, and then with Hoechst 33258 (Wu et al., 2018; Laha et al., 2019; Zhang et al., 2020). Briefly, A3 cells were subjected to 6.25, 12.5, and 25 μM DN for 48 h for fluorescence microscopy. For AO/EB staining, cells were harvested and washed three times with PBS. Subsequently, the cells were stained with 0.1 ml of 4 μg/ml AO/EB solution (Sigma-Aldrich Corp., St. Louis, MO, USA) at 37°C in the dark for 15 min. For MDC labeling, resuspended cells were incubated with fluorescent dye at 37°C in the dark for 45 min. The cells were centrifuged and resuspended with 100 μl of assay buffer according to the manufacturer’s protocol for the Autophagy Detection Kit (ab139484; Abcam). The remaining A3 cells were fixed with 70% ethanol and incubated with 1 μg/ml Hoechst 33258 solution (Sigma-Aldrich Corp., St. Louis, MO, USA) at room temperature for 10 min. A confocal laser scanning microscope was used to observe the morphology of each group of cells (Leica Microsystems, Hessen Wetzlar, Germany).

### Cellular Ultrastructure Examination

Cellular ultrastructure was examined using conventional transmission electron microscopy (TEM), as described previously (Yuan et al., 2017). Briefly, A3 cells treated with different concentrations of DN (6.25, 12.5, and 25 μM) for 48 h were collected as mentioned in *Cell Cycle Analysis*. The cells were subsequently fixed with 2.5% glutaraldehyde (Analytical grade reagent; Sinopharm Chemical Reagent Co., Ltd, Beijing, China) containing 0.1 M sucrose (Analytical grade reagent; Sinopharm Chemical Reagent Co., Ltd) and 0.2 M sodium cacodylate (Analytical grade reagent; Sinopharm Chemical Reagent Co., Ltd) for 24 h at 4°C, followed by the addition of 10 g/L OsO_4_ (Analytical grade reagent; Sinopharm Chemical Reagent Co., Ltd). The cells were then dehydrated, embedded in epoxy resin, cut into sections, and observed with an H700 transmission electron microscope (Hitachi, Tokyo, Japan).

### RNA Extraction

A3 cells were treated with 12.5 and 25 μM DN for 48 h, while untreated A3 cells served as the control. Total RNA was isolated from each sample using the Cell RNA Kit (Omega Bio-Tek, Inc., Norcross, GA, USA) according to manufacturer’s instructions. The quality and concentration of the RNA were estimated using a NanoPhotometer spectrophotometer (IMPLEN, Westlake Village, CA, USA), and the integrity of the RNA was detected on 1% agarose gels. High-quality RNAs were used for library construction and quantitative analysis.

### Library Construction for Digital Gene Expression Sequencing

For RNA library construction, a total of 5 μg of RNA per sample was used as input to produce sequencing libraries using an NEBNext Ultra RNA Library Prep Kit for Illumina (NEB, Ipswich, MA, USA) according to the manufacturer’s recommendations. During the process of library construction, a unique barcode was added to each sample to distinguish between the different samples. Briefly, we isolated and purified mRNA from total RNA using poly-T oligo-attached magnetic beads. The first strand of cDNA was synthesized using random hexamer primer and M-MuLV Reverse Transcriptase H (RNase H) from the fragmented RNAs. Then, the second strand of cDNA was synthesized using DNA polymerase I and RNase H. After repairing the cohesive ends with exonuclease/polymerase and adenylation of 3ʹ ends, a specific adapter was added in each sample through amplification. Finally, PCR products of length ranging from 150 bp to 200 bp were purified using AMPure beads (Beckman Coulter, Beverly, MA, USA). The quality and quantity of the final libraries were assessed by using an Agilent Bioanalyzer 2100 (Agilent Technologies, Palo Alto, CA, USA) before sequencing.

### Analysis of Differentially Expressed Genes

After filtering reads containing adapter, reads containing ploy-N, and low-quality reads from raw data, clean data were mapped to the human genome. Then, the number of reads mapped to each gene were calculated using HTSeq v0.6.0 (Illumina Inc, Santa Clara, CA, USA). The differential expression between the DN-treated and control groups was calculated by using the DESeq R package (1.18.0). Their expression levels were measured using fragments per kilobase of transcript of exon model per million reads mapped (FPKM) values. Genes with an adjusted *p*-value <0.05 found using DESeq2 were considered to be differentially expressed.

### Functional Annotation and Gene Ontology (GO)/Kyoto Encyclopedia of Genes and Genomes (KEGG) Enrichment Analysis

For functional annotation of differentially expressed genes, GOseq R package and KOBAS software were used to analyze GO enrichment and KEGG pathways, respectively (Kanehisa and Goto, 2000; Young et al., 2010; Kanehisa et al., 2016).

### Real-Time Quantitative PCR Validation

Total RNA was extracted from the DN treated and control groups, as previously described in 2.8, and reverse-transcribed into cDNA using a PrimeScript™ reverse transcription (RT) reagent Kit (Takara, Otsu, Japan) according to the manufacturer’s instructions. Two other pairs of primers for glyceraldehyde-3-phosphate dehydrogenase (GAPDH) and β-actin were used as internal controls for normalization of gene expression, as shown in **Supplementary File S5**. Primers of 16 differentially expressed genes used for quantitative reverse-transcription polymerase chain reaction (qRT-PCR) analysis were designed using PrimerQuest (https://sg.idtdna.com/PrimerQuest/Home). The qRT-PCR was performed using LightCycler 480 (Roche, Basel, Switzerland). The reaction volume was 20 μl; it contained 10 μl of SYBR mix (#A25741, Thermo Fisher, Waltham, MA, USA), 0.6 μl of each primer, 2 μl of cDNA template, and 6.8 μl of RNase-free water. The thermocycling program was as follows: 95°C for 5 min, followed by 45 cycles of 95°C for 10 s, 57°C for 10 s, and 72°C for 20 s. Relative gene expression levels were calculated by using the 2 ^−ΔΔCt^ method (Zhang et al., 2020).

### Western Blotting

A3 cells were treated with 6.25, 12.5, and 25 μM DN for 48 h, and harvested and washed with cold PBS. Total protein was extracted from RIPA Lysis Buffer (#P0013B, Beyotime, Shanghai, China) with 1% phenylmethyl sulfonylfluoride. The protein concentrations of different samples were determined using the BCA assay, as described previously (Heshmati et al., 2020). Proteins were electrophoresed using 8–15% SDS-PAGE and electrically transferred onto poly (vinylidene fluoride) membranes (ISEQ00010 0.22 μm, Millipore, MA, USA) successively. After blocking with 5% non-fat milk, the membranes were incubated with rabbit anti-AMPK, p-AMPK (Thr172), mTOR, p-mTOR (Ser2448), ATG1, p-ATG1 (Ser555), Beclin-1, p-Beclin-1 (Ser93), and GAPDH antibodies (Cell Signaling Technology, MA, USA) at 4°C overnight. After incubation, membranes were washed with Tris-buffered saline containing 0.1% Tween 20 (TBST) and incubated with horseradish peroxidase-conjugated secondary antibodies or anti-rabbit IgG antibodies for 1.5 h at room temperature. The proteins were visualized using Millipore’s enhanced chemiluminescence (ECL) and detection system (ChemiDoc Touch, BioRad, Germany).

### Statistical Analysis

In our study, three replicates were used for each treatment. Data are shown as means ± standard error (SE) of three replicates. The SPSS 21.0 software package (SPSS, Chicago, IL, USA) was used to detect statistically significant differences among different groups. Differences were considered statistically significant when *p* < 0.05.

## Results

### Isolation of DN From *A. arundinis*

Repeated column chromatography using silica gel, a reversed-phase C_18_ column, and Sephadex LH-20 were used for compound isolation from *A. arundinis* cultures. The chemical structure of DN was determined using mass spectrometry and NMR. DN was isolated as a yellowish oil. Its molecular formula was established as C_20_H_24_O_4_, as evidenced from the quasimolecular ion peak at *m/z* 327.1597 [M − H]^−^ (calcd. for C_20_H_23_O_4_, 327.1602) in its (–)-HRESIMS spectrum. The structure of DN was finally elucidated as a prenylated diphenyl ether by comparison of its NMR data with those reported previously in the literature (Zhang et al., 2020). HPLC analysis indicated that the purity of DN was > 98% (**Figure 1**).

### *In Vitro* Growth Inhibitory Effect of DN

**Figure 2:**
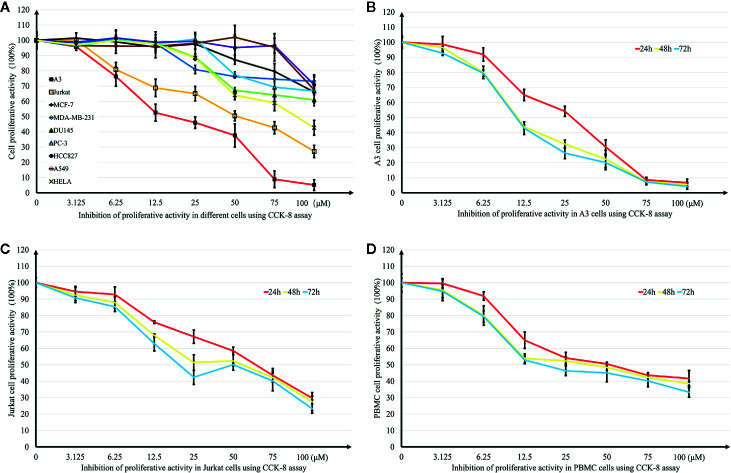
Effects of Diorcinol N (DN) on cancer cell lines. **(A)** Inhibitory effects of DN on nine cancer cell lines (lymphoblastic leukemia cell lines A3 and Jurkat; breast cancer cell lines MCF-7 and MDA-MB-231; prostate cancer cell line DU-145 and PC-3; lung cancer cell line A549 and HCC827); cervical cancer cell line HeLa. **(B–D)** Inhibitory effects of DN on three leukemia cell lines: lymphoblastic leukemia cell lines, A3 and Jurkat, and human peripheral blood mononuclear cells (PBMCs). (n = 5).

Growth inhibition effects induced by DN on different tumor cell lines were investigated using the CCK-8 assay. Six cancer cell lines were treated with different concentrations (0.39–50 μM) of DN for 48 h. DN showed concentration-dependent inhibitory effects on the growth of these different cancer cell lines (**Figure 2A**). However, the cell lines showed different levels of sensitivity to DN. The most remarkable effect was observed for A3 cells (IC_50_ = 16.31 μM) (**Figure 2A**). Since A3 cells are T cell leukemia cells, we examined the sensitivity of different leukemia cell lines to DN. Concentration- and time-dependent growth inhibition was observed for three leukemia cell lines, A3, Jurkat, and PBMCs (**Figures 2B–D**). As A3 cells were the most sensitive to DN compared to PBMCs and Jurkat cells, they were used in subsequent experiments.

### DN Modulated the Transcription of Different Genes in A3 Cells

**Figure 3:**
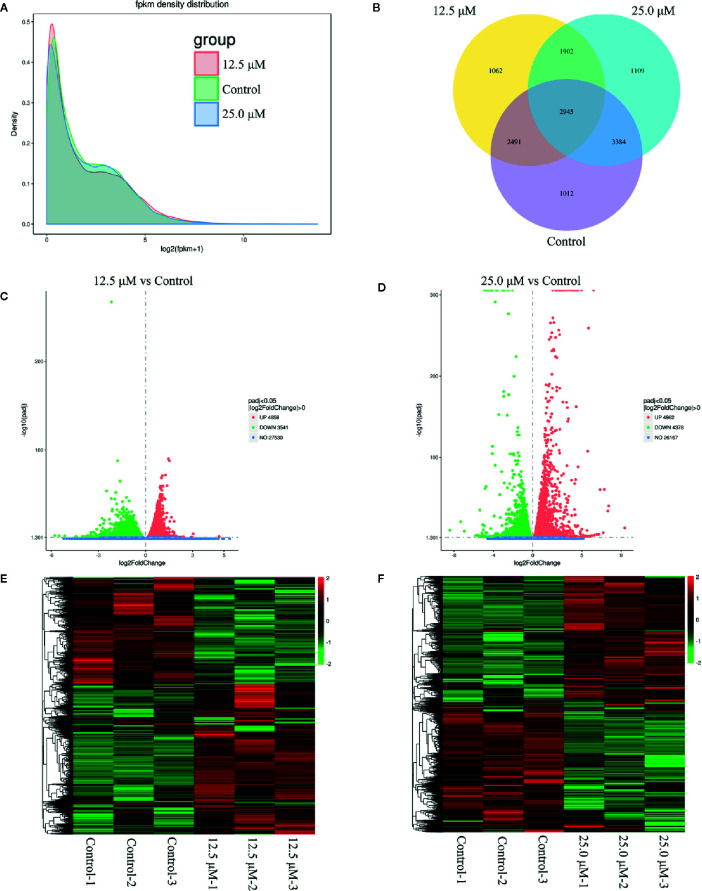
Overview of genes that were differentially expressed between untreated cells and diorcinol N (DN)-treated (12.5 and 25 μM) A3 cells. **(A)** FPKM density distribution. **(B)** Venn diagram of the differentially expressed genes between DN-treated A3 cells and control cells. **(C)** Volcano diagram of the differentially expressed genes between 12.5-μM-DN-treated A3 cells and control cells. **(D)** Volcano diagram of the differentially expressed genes between 25.0-μM-DN-treated A3 cells and control cells. **(E)** Heatmaps of upregulated and downregulated genes in 12.5-μM-DN-treated A3 cells and control cells. **(F)** Heatmaps of upregulated and downregulated genes in 25-μM-DN-treated A3 cells and control cells. FPKM, fragments per kilobase of transcript of exon model per million reads mapped.

To investigate the anticancer mechanism of DN, transcriptional profiling analysis was performed for untreated A3 cells and A3 cells treated with different concentrations of DN. RNA-Seq was used to generate 65,926,953 reads in the 12.5 μM-DN-treated group, 45,756,714 reads in the 25-μM-DN-treated group, and 66,200,048 reads in the control group (**Table S1**). These reads were mapped to the human genome and the unique mapping rates were found to be over 80%, which represented 8400, 9340, and 9832 expressed genes, respectively (**Table S1**). Then, the expression levels of these genes were quantified based on FPKM values (**Figure 3A** and **Table S1**). These results indicated that compared to the control group, 2,964 and 3,011 genes were uniquely expressed in the 12.5-μM-DN- and 25-μM-DN-treated groups, respectively (**Figure 3B**). Using P < 0.05 and a 2-fold change (FC) as the conditions for discrimination, 8400 differentially expressed genes were found in the 12.5-μM-DN-treated group, among which 47.85% (4859) were downregulated and 42.15% (3541) were upregulated (**Figure 3C**, **Tables S2**, **S3**). Moreover, the number of differentially expressed genes rose as the concentration of DN increased (**Figure 3D**, **Tables S4**, **S5**). To assess the expression patterns of mRNAs at different concentrations of DN, heatmaps were used to determine overall transcriptomic differences. The heatmaps showed accurate repeatability and high reliability (**Figures 3E, F**).

### GO and KEGG Enrichment Analyses of Differentially Expressed Genes in DN-Treated A3 Cells

**Figure 4:**
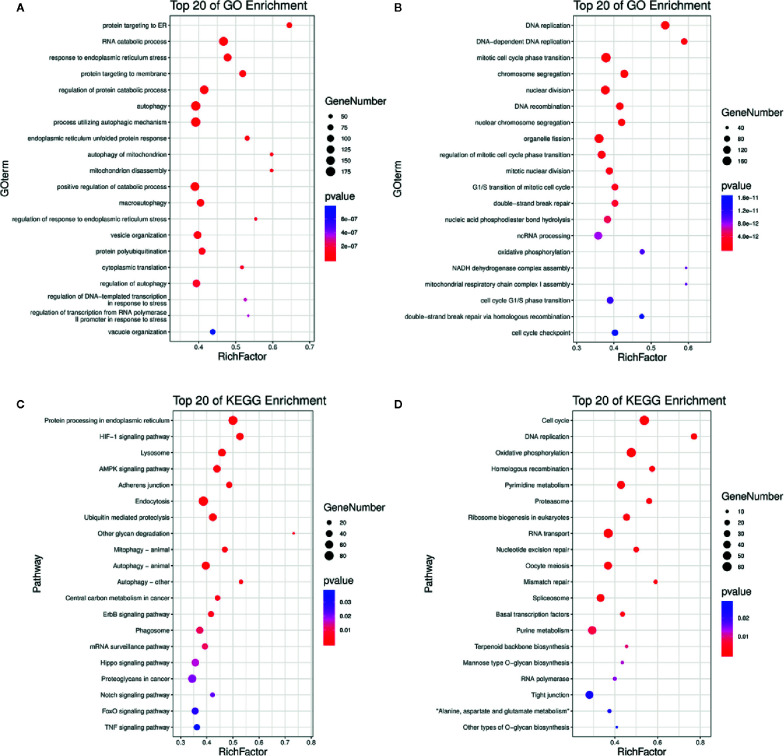
GO and KEGG analyses of differentially expressed genes. **(A, B)** Upregulated and downregulated genes linked with GO enrichment terms. **(C, D)** Upregulated and downregulated genes that were enriched in KEGG pathways. GO, gene ontology; KEGG, Kyoto Encyclopedia of Genes and Genomes; bubble size, the number of genes belonging to this pathway at the target gene concentration; bubble color, significance value of enrichment; RichFactor, the ratio of the number of genes in the pathway entry of the differentially expressed genes to the total number of genes in the pathway entry of all genes.

Based on the *p*-value thresholds described above, the differentially expressed genes were divided into four clusters. Among four clusters, clusters 3 was significantly enriched with 3,413 differentially expressed genes (**Figure S1**). In addition, cluster 2 containing 155 differentially expressed genes was significantly enriched as the upregulated cluster. To estimate the functions of genes that were differentially expressed between the treated and control groups, GO category and KEGG pathway analyses were performed for the differentially expressed genes. The enrichment of GO categories for these genes showed that the upregulated genes were mainly involved in the cellular response to endoplasmic reticulum stress, autophagy, mitochondrion disassembly, protein polyubiquitination, and vacuole organization (**Figure 4A**, **Tables S6**, **S7**), whereas the downregulated genes were mainly related to DNA replication, mitotic cell cycle phase transition, chromosome segregation, G1/S transition of mitotic cell cycle, and cell cycle G1/S phase transition (**Figure 4B**, **Tables S6**, **S7**). Analysis of the metabolic pathways in which these differentially expressed genes participated showed that the most enriched upregulated pathways were involved in adenosine 5’-monophosphate-activated protein kinase (AMPK) signaling, autophagy, epidermal growth factor receptor signaling, phagosome formation, Notch signaling, forkhead box O (FoxO) signaling, and tumor necrosis factor (TNF) signaling (**Figure 4C**, **Tables S8**, **S9**), whereas the most enriched downregulated gene pathways included cell cycle, DNA replication, oxidative phosphorylation, nucleotide excision repair, and mismatch repair (**Figure 4D**, **Tables S8**, **S9**). The effects of DN were most closely associated with the pathways of autophagy (**Figure S2**), cell cycle (**Figure S3**), and DNA replication (**Figure S4**), all of which can decrease cell viability. These findings indicate that DN may modulate A3 cell biological processes by inhibiting the cell cycle and inducing autophagy.

### DN Induced G1/S-Phase Arrest in A3 Cells

**Figure 5:**
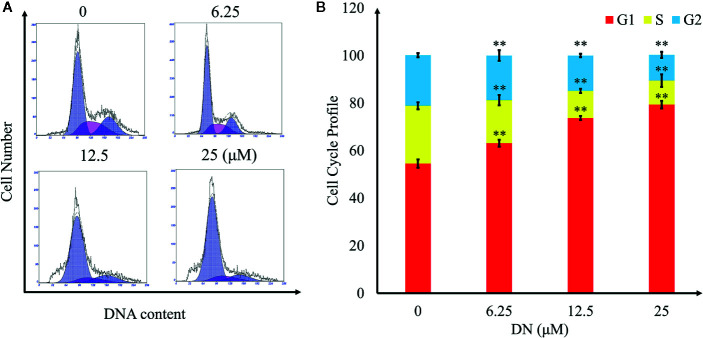
Cell cycle profiling of A3 cells exposed to diorcinol N (DN). **(A)** Cell cycle distribution of DN-treated A3 cells. **(B)** Cell cycle profile of DN-treated A3 cells. **p < 0.01 indicates a significant difference versus the untreated group.

To explore the effects of DN on the cell cycle of A3 cells, cells were treated with 6.25, 12.5, and 25 μM DN for 48 h and analyzed using flow cytometry. As shown in **Figures 5A, B**, treatment with DN significantly increased the cell population in the G1 phase from 54.55% for untreated cells to 79.31% for 25-μM-DN-treated A3 cells. Additionally, DN-treated cells showed a reduction in the population in S phase (18.15% (6.25 μM), 11.41% (12.5 μM), and 10.12% (25 μM DN)) from 24.3% for untreated cells, and a reduction in the population in the G2 phase (18.82% (6.25 μM), 14.95% (12.5 μM), and 10.81% (25 μM)) from 21.25% for untreated cells. These findings indicate that DN induced A3 cell cycle arrest in the G1/S phase.

### DN Induced Autophagy in A3 Cells

**Figure 6:**
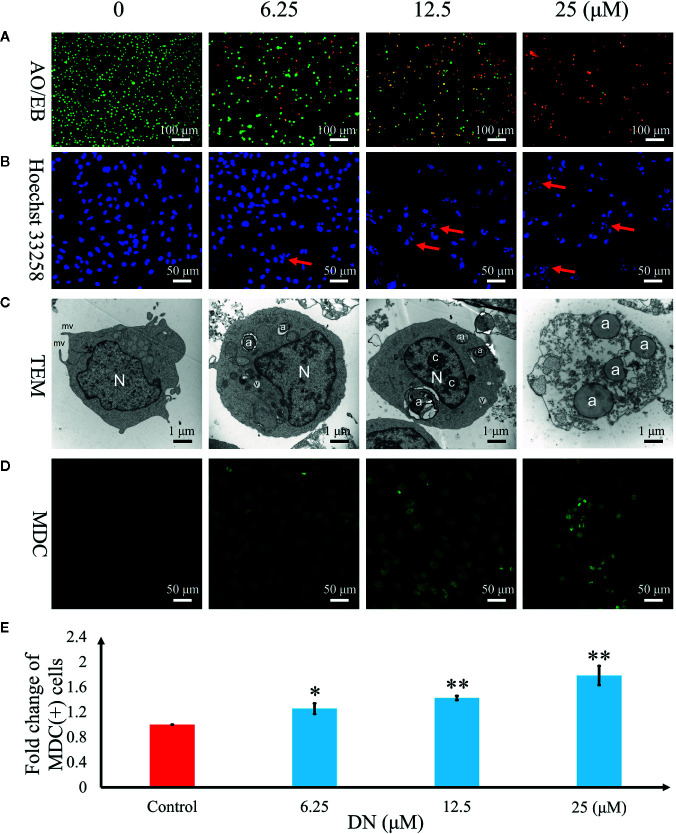
Diorcinol N (DN) induced autophagy in A3 cells. **(A)** Elevated plasma membrane permeability was detected by staining cells in the AO/EB double-staining assay. Original magnification, ×100. **(B)** Autophagy-related nuclear morphological changes were detected by labeling cells with Hoechst 33258. Autophagic cells were defined as those with blue nuclei that exhibited a fragmented/condensed appearance (red arrow). Original magnification, ×200. **(C)** Ultrastructural changes in A3 cells were detected via TEM. a, autophagosome; c, condensed chromatin; mv, microvillus; N, nucleus; v, vacuole. **(D)** The autophagosomes of autophagic cells were labeled using MDC and viewed under a fluorescence microscope. Original magnification, ×200. **(E)** Bar graph showing the quantification of MDC-positive A3 cells. Mean values are shown with standard deviation (SD). AO/EB, acridine orange/ethidium bromide; TEM, transmission electron microscopy; MDC, monodansylcadaverine. *p < 0.05, **p < 0.01 indicates a significant difference versus the untreated group.

To further examine the mechanism of anticancer activity in DN-treated A3 cells, cytological changes were observed using fluorescence microscopy and TEM. The results indicated that A3 cells showed dose-dependent autophagy-like cytological changes (**Figure 6**) after exposure to DN at doses ranging from 6.25 to 25 μM. The observed morphological features included elevated permeability (**Figure 6A**), atypical chromatin (**Figure 6B**), ultrastructural disorganization (**Figure 6C**), and autophagic vacuoles (**Figure 6C**). Furthermore, autophagic vacuoles were assessed using imaging studies with the use of MDC staining. Cells with activated autophagy had a green fluorescent signal in the cytoplasm (**Figure 6D**). Increasing MDC fluorescence in A3 cells was observed after DN treatment (**Figure 6E**).

### Validation of the Expression of Autophagy- and Cell Cycle-Associated Genes and Proteins

**Figure 7:**
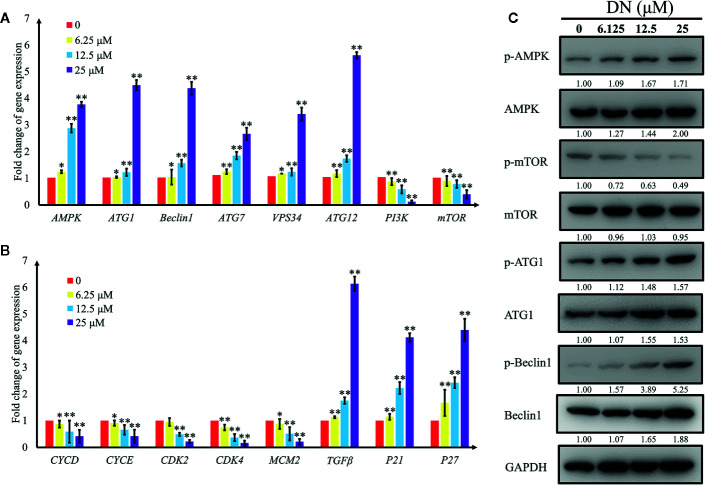
Validation of expression levels of genes related to autophagy and the cell cycle. **(A)** Differentially expressed autophagy-related genes induced by diorcinol N (DN). **(B)** Differentially expressed cell cycle-related genes induced by DN. **(C)** Western blot analysis of AMPK, p-AMPK (Thr172), mTOR, p-mTOR (Ser2448), ATG1, p-ATG1 (Ser555), Beclin1, p-Beclin1 (Ser93) expression A3 cells treated with different concentrations of DN for 48 h. *p <0.05, **p <0.01 indicates a significant difference versus the untreated group.

To validate the results of the RNA-Seq analysis, qRT-PCR was performed to validate the expression patterns of 16 differentially expressed genes, which are closely related to autophagy and the cell cycle (**Table S10**). We measured the levels of expression of genes that are involved in autophagy—adenosine 5’-monophosphate (AMP)-activated protein kinase (*AMPK*), autophagy-related gene 1 (*ATG1*), *beclin-1*, vacuolar protein sorting 34 (*VPS34*), autophagy-related gene 7 (*ATG7*), autophagy-related gene 12 (*ATG12*), phosphoinositide 3-kinase (*PI3K*), and mammalian target of rapamycin (*mTOR*)—and in the cell cycle—cyclin D (*CycD*), cyclin E (*CycE*), cyclin-dependent kinase 2 (*CDK2*), cyclin-dependent kinase 4 (*CDK4*), minichromosome maintenance complex component 2 (*MCM2*), transforming growth factor beta (*TGFβ*), cyclin-dependent kinase inhibitor 1α (*p21*), and cyclin-dependent kinase inhibitor 1β (*p27*). The results indicate a significant increase in the expression of genes involved in the promotion of autophagy (*AMPK, beclin-1, ATG1, VPS34*, and *ATG7*) and a reduction in the expression of genes involved in the inhibition of autophagy (*PI3K* and *mTOR*) (**Figure 7A**). Additionally, we detected a significant decrease in *CycD, CycE, CDK2, CDK4*, and *MCM2* transcript levels; these genes play positive roles in the cell cycle, whereas cell cycle inhibitory genes, such as transforming growth factor beta (*TGFβ*), *p21* and *p27*, were upregulated (**Figure 7B**). In addition, AMPK levels of expression and the concentrations of its substrates, beclin-1 protein levels, increased following treatment with DN in a dose-dependent manner (**Figure 7C**). Activated AMPK negatively regulates mTOR and, in turn, enhances autophagy flux. In our experiments, we observed that DN dephosphorylated mTOR in a dose-dependent manner (**Figure 7C**). Finally, DN also induced a dose-dependent increase in the levels of phosphorylated ATG1, which is a downstream target of mTOR (**Figure 7C**).

## Discussion

While tremendous progress has been made in the treatment of pediatric ALL, the success rate of current treatments is much more modest in adults (Aldoss and Stein, 2018). Hence, new therapeutic agents are urgently needed for ALL therapy (Thu Huynh and Bergeron, 2017; Dyczynski et al., 2018a). The antifungal, anticancer, and antinociceptive effects of diorcinol have been investigated in previous studies (Gao et al., 2013; Li et al., 2015; Zhuravleva et al., 2016; Li et al., 2018; Zhang et al., 2018). However, the mechanism by which DN exerts anticancer effects on ALL cells requires further elucidation. In this study, we provide evidence that DN inhibited growth and induced autophagy in leukemia cells by modulating multiple cell signaling molecules. To characterize the anticancer mechanism of DN in human cell lines, we first explored the effects of DN on the viability of human cancer cell lines and PBMCs. DN decreased the viability of numerous cancer cell lines in our present study; however, the most obvious anticancer effect of DN was observed on A3 cells in the CCK-8 assay. Digital expressed gene profiling was performed to identify genes that were differentially expressed in untreated and DN-treated A3 cells. GO and KEGG analyses were performed for the 9,340 differentially expressed genes observed in treated and untreated A3 cells. We found that DN could inhibit the growth of A3 cells by affecting biological processes, such as autophagy, cell cycle, DNA replication, vacuole organization, and mitotic nuclear division. However, we detected that the genes associated with the apoptotic pathway were not in the top 20 enrichment list. Thus, DN probably modulated the expression of genes related to autophagy and the cell cycle to a greater degree. The effects of DN on A3 cells were similar to those observed for Ghrelin on Jurkat and Molt-4 human lymphoblastic cell lines (Heshmati et al., 2020).

Studies have reported changes in the expression of genes related to the cell cycle that are relevant to abnormal karyomorphism. Hoechst 33258 labeling revealed DN-induced pyknotic karyomorphism over time. Pyknotic karyomorphism has been found during autophagy in other leukemia cells and is induced by treatment with different kinds of anticancer drugs (i.e., 786-O cells and L-02 cells) (Yuan et al., 2017; Chen et al., 2018). The fidelity of replication is affected by abnormal karyomorphism, and low fidelity of replication could lead to cell cycle arrest (Dyshlovoy et al., 2020; Oh et al., 2020). Our findings indicate that DN arrested the cell cycle of A3 cells at the G1/S phase, which is similar to the effects of previously reported anticancer agents, such as triacanthine and licoricidin (Ji et al., 2017; Shin et al., 2019). In addition, transcriptome profiling and qRT-PCR analyses show that cycle-stimulative genes, such as *CDC, CDE, CDK2*, and *CDK4*, were downregulated in DN-treated A3 cells, whereas cycle-suppressive genes, such as *TGFβ, p21*, and *p27* were upregulated. TGFβ plays a vital role in promoting G1/S phase arrest *via* a complex of mothers against decapentaplegic homolog 2/3/4 and specificity protein 1 (Sun et al., 2019). Phosphorylated tyrosine-containing p27 inhibits CDK4 and CDK2 to cause cell cycle G1/S arrest (Kang et al., 2002; Blain, 2018). As a cyclin-dependent kinase (CDK) inhibitor, p21 binds to cyclin A/CDK2, E/CDK2, D1/CDK4, and D2/CDK4 complex to inhibit the phosphorylation of retinoblastoma protein and thus, inhibit G1/S transition (Kang et al., 2002; Karimian et al., 2016). As a new marker for proliferating cells, MCM2 is one of the six highly conserved proteins that form a double hexameric MCM complex that is essential for the pre-replication apparatus and may be involved in the formation of replication forks for recruiting other DNA replication-related proteins in the G1 phase (Braun and Breeden, 2007). The results show that the downregulated genes are closely related to the cell cycle and thus influence the viability of A3 cells, which is also supported by the results of the Hoechst 33258 staining and cell cycle analysis. The cellular results, together with gene expression, indicate that DN-treated leukemia cells cannot progress through the G1/S checkpoint, which is consistent with previous findings (Vijayaraghavan et al., 2017). Previous studies have shown that the cell cycle is tightly controlled by precise mechanisms, among which autophagy is an important upstream regulator of the cell cycle (Mathiassen et al., 2017; Zheng et al., 2019). Therefore, the observed cell cycle arrest, combined with reduced cell viability, caused by DN in A3 cells could explain the autophagy-inducing effect of DN on A3 cells.

Literature evidence identifies autophagy as an important mode of cancer treatment, *via* the promotion of cell cycle arrest (Onorati et al., 2018; Kocaturk et al., 2019). Autophagic cells present characteristics, such as elevated plasma membrane permeability, karyopyknosis, and autophagic body formation (Zhang et al., 2015; Karch et al., 2017; Padman et al., 2017). Therefore, plasma membrane permeability, autophagosomes, karyomorphism, and ultrastructure of DN-treated A3 cells were examined by labeling with AO/EB, Hoechst 33258, and MDC, as well as TEM, respectively, to detect the effects of DN on autophagy. Our results indicate that DN increased the plasma membrane permeability of A3 cells. Previous studies have reported that anticancer agents, such as α-hederin and curcumin, have a similar effect on the permeability of cell membranes (Lorent et al., 2016; Seo et al., 2018). DN has been shown to induce typical autophagic changes in A3 cells, such as cell structure disorder, vacuolation, and autophagosome formation. Moreover, we observed that the increased expression of AMPK at the gene and protein levels, which can directly or indirectly suppress the expression of downstream mTOR (Jia et al., 2019). Downregulated *mTOR* can promote autophagy *via* the formation of the ATG1-ATG13-FIP200 complex, which causes the upregulation of *p21* and *p27*, leading to cell cycle arrest in the G1 phase (Jung et al., 2018; Kazemi et al., 2018). In addition, transcriptional levels of *PI3K*, a negative regulator of autophagy, had decreased, resulting in the downregulation of downstream *mTOR via* the PI3K/ATK/mTOR signaling pathway (Chen et al., 2019). Moreover, beclin-1 is phosphorylated by ATG1 and forms the Beclin-1-VPS34 complex to promote the localization of autophagic proteins in autophagic vesicles (Dyczynski et al., 2018b; Feng et al., 2019). ATG7 acts as an E1-like ubiquitin-activating enzyme to activate ATG12. Subsequently, the activated ATG12 forms a complex with ATG5 and ATG16 to promote the process of autophagy. Our results also show that DN induced autophagy by increasing the expression of autophagy-related genes such *ATG1, ATG7, ATG12, Beclin-1*, and *VPS34* (Dyczynski et al., 2018b; Sun et al., 2018; Wang et al., 2018). Overall, our data support the conclusion that DN regulated gene expression to induce autophagy, which is the main mechanism underlying its anticancer effects in A3 cells.

The fungal metabolite DN exerts its effects against A3 leukemia cells by decreasing their viability, altering plasma membrane permeability, promoting autophagic morphological changes, arresting the cell cycle at the G1/S phase *via* p21, p27, TGF-β, and the PI3K/Akt/mTOR pathways. Although further study is required to investigate the anticancer activity of DN, our study demonstrates the promise of DN as a lead or even a candidate molecule for the treatment of acute lymphoblastic leukemia.

## Data Availability Statement

The datasets for this study can be found in the NCBI sequence read archive (https://www.ncbi.nlm.nih.gov/bioproject/637910).

## Author Contributions

PZ and X-LY designed experiments. X-QL and KX carried out experiments. X-ML analyzed experimental results. LX, X-QL, KX, and X-ML analyzed and interpreted the data. X-LY drafted and revised the manuscript. All authors contributed to the article and approved the submitted version

## Funding

This work was supported by the Natural Science Foundation of China (grant number 31700295), the Science Foundation for Young Scholars of Institute of Tobacco Research of Chinese Academy of Agricultural Sciences (grant number 2020A01), and the Agricultural Science and Technology Innovation Program (Grant No. ASTIP-TRIC05). The authors declare that this study received funding from the Key Projects of Wannan Tobacco Co., Ltd. (grant no. 20180023). The funder was not involved in the study design, collection, analysis, interpretation of data, the writing of this article or the decision to submit it for publication.

## Conflict of Interest

Author LX was employed by the company Wannan Tobacco Group Co., Ltd.

The remaining authors declare that the research was conducted in the absence of any commercial or financial relationships that could be construed as a potential conflict of interest.

## Abbreviations

ALL, Acute Lymphoblastic Leukemia; AO/EB, Acridine Orange/Ethidium Bromide; AMPK, Adenosine 5’-monophosphate (AMP)-Activated Protein Kinase; ATG, Autophagy-Related Gene; CCK-8, Cell Counting Kit-8; CDK, Cyclin-Dependent Kinase; CycD, Cyclin D; CycE, Cyclin E; DMSO, Dimethyl Sulfoxide; DN, Diorcinol N; EtOAc, Ethyl Acetate; FBS, Fetal Bovine Serum; FC, Fold Change; FCS, Fetal Calf Serum; FoxO, Forkhead Box; FPKM, Fragments Per Kilobase Of Transcript Of Exon Model Per Million Reads Mapped; GAPDH, Glyceraldehyde-3-Phosphate Dehydrogenase; GO, Gene Ontology; HPLC, High Performance Liquid Chromatography; HRESIMS, High Resolution Electrospray Ionization Mass Spectrometry; IC_50_, The Half Maximal Inhibitory Concentration; KEGG, Kyoto Encyclopedia of Genes and Genomes; MCM2, Minichromosome Maintenance Complex Component 2; MDC, Monodansylcadaverine; MEM, Minimum Essential Medium; MeOH, Methanol; mTOR, Mammalian Target of Rapamycin; NMR, Nuclear Magnetic Resonance; p21, Cyclin-Dependent Kinase Inhibitor 1α; p27, Cyclin-Dependent Kinase Inhibitor 1β; PBMCs, Peripheral Blood Mononuclear Cells; PBS, Phosphate-Buffered Saline; PE, Petroleum Ether; PI3K, Phosphoinositide 3-Kinase; qRT-PCR, Quantitative Reverse-Transcription Polymerase Chain Reaction; RNase H, Reverse Transcriptase H; ROS, Reactive Oxygen Species; RP, Reverse Phase; RPMI, Roswell Park Memorial Institute; RT, Reverse Transcription; SE, Standard Error; TBST, Tris-buffered saline containing 0.1% Tween 20; TEM, Transmission Electron Microscopy; TGFβ, Transforming Growth Factor Beta; TNF, Tumor Necrosis Factor.
